# Review of "Scaffolding in Tissue Engineering", by Peter X. Ma and Jennifer Elisseeff (Editors)

**DOI:** 10.1186/1475-925X-5-52

**Published:** 2006-10-03

**Authors:** Eduardo Abreu

**Affiliations:** 1 Childrens' Hospital of Boston, Department of Orthopaedic Surgery, Enders 1022, 300 Longwood Street, Boston MA 02115, USA

Tissue engineering (TE) is a relatively new, interdisciplinary and multidisciplinary field that has seen intense development in recent years. One of the main motivations for TE research is the chronic shortage of organ donors and other limitations related to organ and tissue transplantation. The idea that tissues, and ultimately organs, can be "engineered" to be used in patients requiring transplantation is at the same time revolutionary and stimulating. However, TE is a discipline still at its infancy, an intricate puzzle far from being completed. One key piece of this puzzle is the scaffold that provides temporary support for cell proliferation and differentiated function, allowing neotissue formation and initial remodeling. Surely, the choice of the best scaffold for a particular TE application and how to change an existing scaffold to improve its performance are important components of any TE research project.

Despite the significance of scaffolds for TE, until recently no book had been entirely dedicated to this subject. Having noticed this void, Peter X. Ma and Jennifer H. Elisseeff decided to put together a stellar team of international contributors to bring into existence "Scaffolding in Tissue Engineering". Scaffolding is a complex subject and a dynamic area of research, which involves the interaction of various areas of knowledge, such that new scaffolds are continually being developed, fabricated, improved, transformed and evaluated. Hence, it could be expected that the preparation of a comprehensive book about scaffolding in TE and, most of all, the coordination of the contributions of a large group of experts in TE (but with different backgrounds) would be an arduous task. All these efforts resulted in an extraordinary book. As a consequence, the comments in this review should be seen as positive criticisms and, who knows, suggestions for future editions of the book.

"Scaffolding in Tissue Engineering", an encyclopedic book 624 pages long (not including the index at the end), is divided into 38 chapters written by 82 contributors. The chapters are arranged into four parts: scaffolding materials, scaffold fabrication technologies, materials modifications and properties and tissue engineering applications. Most certainly, the editors attempted to make the book more didactic by classifying and grouping the chapters into four different areas of a subject that is difficult to be compartmentalized. For example, it may be sometimes hard to demarcate where fabrication ends and modification begins.

After reading "Scaffolding in Tissue Engineering", the basic conclusion is that all chapters are individually very well and carefully written, with plenty of helpful pictures, graphs and tables, and have a comprehensive list of references at the end. The chapters are objective and relatively short and this book will be certainly useful to beginners as well as to senior researchers in the field. This level of quality should not be a surprise taking into consideration the excellence of the authors chosen to write the chapters. However, although every chapter is outstanding by itself, when put together somehow they did not mingle well and, as a whole, "Scaffolding in Tissue Engineering" has, in our understanding, some problems in terms of organization.

It is important to emphasize again that because of the complex nature of the subject, to merge so many chapters about different aspects of scaffolding into a book is not a trivial task. An example of how individual contributions may not work as well when brought together is that many chapters begin with a general introduction about the relevance of TE. In our opinion, this kind of introduction makes more sense for individual, not combined, texts. In a book with 38 chapters perhaps it would have been better to summarize the individual contributors' views about TE in a small, initial chapter or introduction. On the other hand, we have to respect the editors' choice in this regard and someone may argue that the original way works better. Another example, not all contributors included at the end of their chapters a section about the future directions of their research. Again in our opinion, it is beneficial to know not only the state of the art of a certain subfield of TE, but also how the researchers see their work progressing in the future. Maybe at another opportunity the editors could kindly suggest to all contributors to keep in mind the importance of discussing future directions for the different TE topics.

Looking into more specific segments of the book, the first part discusses different materials, synthetic or of biological origin, that are used as scaffolds or as part of scaffolds. Examples of these materials are collagen-GAG copolymers, alginates, chitosan, dextran, fibrin and poly(ortho)esters. In the case of poly(ortho)esters only the latest family (poly(ortho)esters IV) is discussed but appropriate bibliography is suggested for other families of poly(ortho)esters. Already at this point, our impression was that some chapters seemed to be misplaced in the book. For example, chapter 4 is dedicated to the role of gelatin in the release of growth factors for TE. However, we believe that the release of growth factors would be more suitably presented as a TE application or part of an application. Chapter 5 also includes examples of scaffold applications in TE (e.g., bone regeneration). Chapter 6 focuses on the photopolymerization of hydrogels, which we believe is a modification of the original scaffold. In our opinion the material presented in chapter 6 would be better suited for part III. Another example, alginates are discussed on chapters 2, 3 and 21 and it is especially difficult to understand why it appears at two consecutive chapters in part I.

The second part of the book focuses on scaffold fabrication technology, that is, how to change scaffold characteristics that can be controlled such as porosity, incorporation of bioactive substances, etc., to improve functionality and performance. Examples of these fabrication technologies that will allow the scaffold to perform many of the critical functions required from the scaffolds are salt leaching, polymer phase separation, freeform fabrication, gas foaming and others. This part of the book also discusses strategies to improve the scaffold delivery and function, such as the development of injectable systems and immunoisolation. Injectable systems, presented only as an application in cartilage TE but that could be very well applied to other areas, are important processes through which the trauma that follows direct scaffold implantation can be minimized. Immunoisolation is an interesting fabrication technology (or modification?) that allows the bidirectional diffusion of nutrients and other substances secreted by encapsulated transplanted cells while preventing the host immune defense to attack the transplanted cells.

In the third part of the book, chapters 16 through 23 describe how materials at first not suitable to be used as TE scaffolds can be turned into usable scaffolds and how some modifications can improve the functionality of existing scaffolds. Among others, the case of the alginates, originally of uncontrollable degradation and lack of cellular interactions, that can be modified (e.g., by cross-linking) so that it can be used in TE applications, is presented. Another modification that can be made is the attachment of peptides with adhesion function to hydrogel systems makes them bioactive scaffolds. Also, the scaffolds' surfaces can be grafted with albumin to make them thromboresistant and more biocompatible. Chapter 20 is dedicated to albumin modification and its use in TE applications. Chapter 16 describes how a thermally induced phase separation technique (TIPS) can be applied to fabricate hydroxyapatite (HAP) based scaffolds (it seems that this chapter should be in part II of the book).

The final part of the book is dedicated to TE applications. A wide range of tissues targeted for TE substitutes are described and discussed in the last 15 chapters of the book. TE applications for orthopaedic tissues (tendons and ligaments, cartilage, meniscus), genitourinary tissue, cornea, hepatic tissue among others are discussed. Also, the role of stem cells and the use of bioreactors in TE are the subject of two of the chapters. In this part of the book we missed a chapter specifically dedicated to bone TE, although some discussion about this particular application appears here and there throughout the book. Moreover, we missed information referring to TE applications that use platelet-rich-plasma (PRP) to functionally improve scaffolds, especially in bone regeneration [1] and as part of novel strategies to improve soft musculoskeletal tissue regeneration [2].

According to the editors, "this book is not only intended to be a textbook for advanced undergraduate and graduate students, but also a review and reference book for researchers in materials sciences, biomaterials, tissue engineering, biotechnology, biomedical engineering, biomedical sciences and clinical laboratories". The editors certainly succeeded in their purpose to produce a robust and outstanding book. Despite minor problems of organization, "Scaffolding in Tissue Engineering" brought together an extraordinary amount of information related to the use of scaffolds in TE. It is unquestionably destined to be a classic book that deserves a place on the shelves of every researcher in TE and libraries.

Congratulation to the editors and to all contributors for this exceptional book.
